# Pregnancy outcomes among HIV-infected women who conceived on antiretroviral therapy

**DOI:** 10.1371/journal.pone.0199555

**Published:** 2018-07-18

**Authors:** Elizabeth M. Stringer, Michelle A. Kendall, Shahin Lockman, Thomas B. Campbell, Karin Nielsen-Saines, Fred Sawe, Susan Cu-uvin, Xingye Wu, Judith S. Currier

**Affiliations:** 1 Division of Maternal Fetal Medicine, Department of Obstetrics and Gynecology, University of North Carolina, Chapel Hill, NC, United States of America; 2 Center for Biostatistics in AIDS Research, Harvard T.H. Chan School of Public Health, Boston, MA, United States of America; 3 Brigham and Women’s Hospital and Harvard T.H. Chan School of Public Health, Boston, MA, United States of America; 4 Division of Infectious Diseases, Department of Medicine University of Colorado School of Medicine, Aurora, CO, United States of America; 5 Division of Infectious Diseases, Department of Pediatrics David Geffen School of Medicine at UCLA, Los Angeles, CA, United States of America; 6 Kenya Medical Research Institute (KEMRI), United States Army Medical Research Directorate-Kenya (USAMRD-K), Henry Jackson Foundation Medical Research International (HJFMRI), Kericho, Kenya; 7 Director, Providence/Boston Center for AIDS Research, Director, Brown Global Health Initiative, Brown University, Providence, RI, United States of America; 8 Division of Infectious Diseases, David Geffen School of Medicine at UCLA, Los Angeles, CA, United States of America; University of New South Wales, AUSTRALIA

## Abstract

As antiretroviral therapy (ART) expands in resource-limited settings, understanding the impact of ART on pregnancy outcomes is critical. We analyzed women who became pregnant on ART while enrolled in a clinical trial (HPTN 052, ACTG A5208, and ACTG A5175); the majority of women were from Africa, with a median age of 29 years. Eligible women were on ART at conception and had a documented date of a last menstrual period and a pregnancy outcome. The primary outcome was non-live birth (stillbirth; spontaneous abortion; elective termination; or ectopic pregnancy) versus live birth. Preterm birth (<37 weeks completed gestation) was a secondary outcome. We used Cox proportional hazards regression models with time-varying covariates. 359 women became pregnant, of whom 253 (70%) met inclusion criteria: 127 (50%) were on NNRTI-based ART, 118 (47%) on PI-based ART, and 8 (3%) on 3-NRTIs at conception. There were 160 (63%) live births (76 term and 84 preterm), 11 (4%) stillbirths, 51 (20%) spontaneous abortions, 28 (11%) elective terminations, and 3 (1%) ectopic pregnancies. In multivariable analysis adjusted for region, parent study, and pre-pregnancy ART class, only older age was associated with increased hazard of preterm birth [HR: 2.49 for age 25–30 years; 95% CI: 1.18–5.26; p = 0.017]. Women conceiving on ART had high rates of preterm birth and other adverse pregnancy outcomes. Despite the benefits of ART, studies designed to investigate the effects of preconception ART on pregnancy outcomes are needed.

## Introduction

Women of reproductive age comprise more than half of all people living with HIV infection worldwide and it is estimated that more than 1.5 million women with HIV will give birth annually[1]. Antiretroviral clinical trials that are not specifically designed for pregnant populations have generally excluded women who are pregnant or who express a desire to become pregnant. Because of this, there are limited data on pregnancy rates and outcomes among women who conceive while on antiretroviral therapy (ART), especially in resource-limited settings (RLS).

Starting in 2013, the World Health Organization (WHO) and UNICEF recommended that all pregnant HIV-infected women commence ART in pregnancy regardless of CD4+ cell count and continue ART for life[2]. Given the subsequent additional WHO recommendation of universal ART for all persons with HIV,[3] an increasing number of women are becoming pregnant while taking ART. As vertical transmission rates continue to decline worldwide, attention has more recently turned to the relationship between exposure to ART during pregnancy and adverse pregnancy outcomes, including low birth weight and/or preterm birth[4–9]. Until recently, few large prospective studies have examined the pregnancy outcomes of women who either conceived while taking ART or commenced ART in pregnancy[10,11]; data are particularly sparse from RLS. Virtually no prospectively and carefully collected data exist on pregnancy outcomes among women who conceive on ART.

A recent meta-analysis of 11 studies that included 19,189 mother-infant pairs found a higher rate of preterm birth among women starting ART prior to conception when compared to those starting ART in pregnancy[12]. Other studies have also suggested that certain antiretroviral regimens may be associated with adverse infant outcomes[13,14]. As the availability of ART expands in RLS, it is critical to understand the impact these drugs may have on maternal and infant outcomes, especially for women whose fetuses are exposed to ART at conception or early in gestation. We sought to describe pregnancy outcomes and factors associated with successful pregnancy outcomes among women who conceived while on ART while prospectively followed in the context of interventional ART trials conducted in RLS.

## Material and methods

In this exploratory analysis, we combined data across three randomized clinical trials of HIV prevention or treatment conducted by the US National Institutes of Health, Division of AIDS Clinical Trials Networks, and the National Institute of AIlergy and Infectious Diseases in resource-limited settings. The deidentified dataset has been provided. (S2 Table) These are AIDS Clinical Trials Group (ACTG) A5208, ACTG A5175, and HIV Prevention Trials Network (HPTN) 052. Briefly, ACTG A5208 was a trial of a protease inhibitor (PI, lopinavir/ritonavir (LPV/r))-based regimen versus a non-nucleoside reverse transcriptase inhibitor (NNRTI, nevirapine (NPV))-based regimen among women with and without prior exposure to single dose nevirapine[15]. ACTG A5175 was a trial of a once-daily PI (atazanavir (ATV), didanosine-EC (DDI-EC) and emtricitabine (FTC))-based regimen versus a once-daily NNRTI (efavirenz (EFV), FTC, and tenofovir disoproxil fumarate (TDF))-based regimen versus a twice-daily regimen (EFV, lamivudine (3TC), zidovudine (ZDV)) for initial treatment of HIV infection[16]. HPTN 052 was a trial of immediate versus delayed ART (EFV, NVP, ATV, LPV/r, and other select ARVs) to prevent sexual transmission of HIV-1 among serodiscordant couples[17]. Pregnancy (or desire for pregnancy during the study period) was an exclusion criterion for entry into each of the studies; however, a number of women became pregnant while on study. Women in A5208 who became pregnant were continued on study drug, but switched from a TDF-containing regimen to a ZDV-containing regimen, and from EFV to NVP or another antiretroviral. Women who become pregnant while on A5175 were allowed to continue on study-provided ART, except women who were taking EFV were required to substitute a different antiretroviral drug such as NVP. All three protocols asked women to recall the date of their last menstrual period at each study visit, and documented their method of contraception. In A5208, women underwent urine pregnancy tests every 4 weeks while on EFV; otherwise, testing was as indicated. Women in A5175 and HPTN 052 underwent urine pregnancy tests every 8 and 4 weeks, respectively. The estimated due date (EDD) and pregnancy data were obtained from patient self report as well as available prenatal records and was recorded onto standardized data collection forms.

We aggregated data from all women enrolled in the three clinical trials who became pregnant during the study period. Women were included in the analysis if a last menstrual period and a pregnancy outcome (included a date of delivery/surgery and birth outcome) were documented and if they were on ART prior to conception. We examined demographic information, maternal HIV viral load prior to pregnancy and during gestation, CD4+/CD8+ measurements prior to pregnancy and during gestation, ART regimen prior to pregnancy and during gestation, pre-pregnancy alcohol/illicit drug use, pre-pregnancy body mass index (BMI), pre-pregnancy graded creatinine and hemoglobin values, pre-pregnancy hepatitis B infection status, and pregnancy history.

Our primary outcome measure was time to non-live birth versus live birth, where non-live births included stillbirths (intrauterine fetal death >20 weeks), spontaneous abortions (<20 weeks), elective terminations, and ectopic pregnancies; these were determined in an *a priori* manner. Among live births, preterm birth was defined as a live birth prior to 37 weeks. Pregnancy outcomes were limited to women with at least one pregnancy while enrolled in the parent study, but we only included the outcome of the first pregnancy; twins were counted as a single outcome because the outcomes were concordant. Date of conception was approximated as two weeks after the date of last menstrual period. Because many variables of interest were related in some way to length of follow-up (i.e., longer pregnancies yielded longer exposure), the variables were confounded with pregnancy outcome. This time bias was remedied by using time-updated covariates in Cox proportional hazards regression models to identify predictors of time from date of conception to date of stillbirth, spontaneous abortion, or elective termination, or date of surgery for ectopic pregnancy; live births were censored at date of delivery. The Cox regression models were adjusted for region and parent study. Multivariable models were built using variables with p<0.20 from the univariable regression analysis and reduced using backward elimination. Post hoc (unplanned) Cox regression models were adjusted for region, parent study, and pre-pregnancy ART class and examined associations with maternal age. We summarized demographic variables by parent study and by live birth versus other. Pregnancy outcomes were summarized by pre-pregnancy ART class. We compared groups using Wilcoxon tests for continuous variables and Chi-square or Fisher’s exact tests for categorical data. A two-sided p-value <0.05 was considered statistically significant. The study was approved by institutional review boards or ethics committees at each study site as well as by other local regularly bodies as deemed appropriate (S1 Table). Written informed consent was obtained before enrollment for all participants.

## Results

In our pooled analysis, 359 women became pregnant while enrolled in one of the three studies. Of these, 253 women (70%) met inclusion criteria for our study and were included in the analysis. The most common reasons women were excluded from the analysis were undocumented last menstrual period or pregnancy outcome (n = 53), or no date of pregnancy outcome and no follow-up date (n = 39), and other (n = 14). Women who were excluded from the analysis were more likely to have been in A5175 (p<0.001), older (median age 29 versus 27 years old; p = 0.046), more likely to be from Asia and Central/South America (p<0.001), more likely to have been on NNRTI-based ART (p = 0.01), and had higher median CD4+ cell count upon entry into the parent study (168 versus 146 cells/mm^3^; p = 0.047). Of the 253 women included in our analysis, 114 (45%) were from HPTN 052, 89 (35%) were from A5208, and 50 (20%) were from A5175. Baseline characteristics by parent study are presented in Table 1. The majority of women were African in origin and were taking an NNRTI-based ART regimen prior to pregnancy. Of the women taking an NNRTI-based regimen, 97 (73%) and 30 (23%) were on an EFV-or NVP-based combination, respectively. Another 118 (47%) were taking PI-based ART, and 8 (3%) on 3-NRTIs at conception. The median pre-pregnancy CD4+ cell count was higher among women in HPTN 052 (median 593 cells/mm^3^ [interquartile range (IQR): 441–764 cells/mm^3^] versus 383 [IQR: 292–458] in A5175 and 321 [IQR: 233–445] in A5208). This was expected since HPTN 052 enrolled women with screening CD4+ between 350 and 550 cells/mm^3^, while the screening CD4+ criterion for A5208 and A5175 were <200 and <300 cells/mm^3^, respectively.

**Table 1:** Baseline participant characteristics by parent study.

			Parent study		
		Total	A5175	A5208	HPTN052
Characteristic		(N = 253)	(N = 50)	(N = 89)	(N = 114)
Region	Other Africa	85 (33.6%)	6 (12.0%)	54 (60.7%)	25 (21.9%)
	Malawi	84 (33.2%)	20 (40.0%)	7 (7.9%)	57 (50.0%)
	South Africa	48 (19.0%)	11 (22.0%)	28 (31.5%)	9 (7.9%)
	Asia	19 (7.5%)	7 (14.0%)	0 (0.0%)	12 (10.5%)
	Central/South America	17 (6.7%)	6 (12.0%)	0 (0.0%)	11 (9.6%)
Maternal age at study entry (years)	Median (IQR)	29 (26, 33)	30 (26, 33)	30 (27, 33)	28 (25, 32)
	>30	105 (41.5%)	23 (46.0%)	43 (48.3%)	39 (34.2%)
	25–30	103 (40.7%)	20 (40.0%)	33 (37.1%)	50 (43.9%)
	18–24	45 (17.8%)	7 (14.0%)	13 (14.6%)	25 (21.9%)
Pre-pregnancy ART class	NNRTI-based ART	127 (50.2%)	30 (60.0%)	35 (39.3%)	62 (54.4%)
	PI-based ART	118 (46.6%)	19 (38.0%)	47 (52.8%)	52 (45.6%)
	NRTIs only ART	8 (3.2%)	1 (2.0%)	7 (7.9%)	0 (0.0%)
Pre-pregnancy CD4 count (cells/mm^3^)	N	241	50	89	102
	Median (IQR)	431 (304, 608)	383(292, 458)	321 (233, 445)	593 (441, 764)
Pre-pregnancy CD8 count (cells/mm^3^)	N	123	34	89	0
	Median (IQR)	748 (550, 980)	676 (567, 881)	785 (550, 1,016)	. (., .)
Pre-pregnancy HIV RNA suppressed to below 400 copies/mL	N	250	50	89	111
	Yes	205 (82.0%)	40 (80.0%)	71 (79.8%)	94 (84.7%)
	No	45 (18.0%)	10 (20.0%)	18 (20.2%)	17 (15.3%)
Pre-pregnancy HIV RNA among those not suppressed (log_10_copies/mL)	N	45	10	18	17
	Median (IQR)	3.8 (3.0, 4.6)	3.5 (2.8, 3.9)	3.8 (3.2, 4.6)	3.8 (2.9, 4.6)
Pre-pregnancy hemoglobin grade	Normal/Mild	243 (96.0%)	48 (96.0%)	83 (93.3%)	112 (98.2%)
	Moderate	5 (2.0%)	1 (2.0%)	3 (3.4%)	1 (0.9%)
	Severe	5 (2.0%)	1 (2.0%)	3 (3.4%)	1 (0.9%)
Pre-pregnancy creatinine grade	N	139	50	89	0
	Normal/Mild	138 (99.3%)	49 (98.0%)	89 (100.0%)	0 (.%)
	Severe	1 (0.7%)	1 (2.0%)	0 (0.0%)	0 (.%)
Pre-pregnancy body mass index (kg/m^2^)	N	139	50	89	0
	Median (IQR)	23.5 (20.5, 26.8)	23.2 (19.6, 26.3)	23.7 (20.7, 26.8)	. (., .)
Pre-pregnancy hepatitis B infection	N	30	9	21	0
	No	27 (90.0%)	8 (88.9%)	19 (90.5%)	0 (.%)
	Yes	3 (10.0%)	1 (11.1%)	2 (9.5%)	0 (.%)
Pre-pregnancy use of alcohol or illicit drugs	N	158	45	0	113
	No	148(93.7%)	40 (88.9%)	0 (.%)	108 (95.6%)
	Yes	10 (6.3%)	5 (11.1%)	0 (.%)	5 (4.4%)
Number of previous pregnancies	N	246	50	88	108
	0	9 (3.7%)	3 (6.0%)	6 (6.8%)	0 (0.0%)
	1–2	128(52.0%)	25 (50.0%)	50 (56.8%)	53 (49.1%)
	≥3	109(44.3%)	22 (44.0%)	32 (36.4%)	55 (50.9%)
Number of pregnancies per woman during the study period	1	225(88.9%)	46 (92.0%)	82 (92.1%)	97 (85.1%)
	2	26 (10.3%)	4 (8.0%)	6 (6.7%)	16 (14.0%)
	3	2 (0.8%)	0 (0.0%)	1 (1.1%)	1 (0.9%)

HPTN 052 did not collect height and weight (for body mass index), CD8+ cell count, or hepatitis B serology; creatinine grading depends upon weight and is thus also missing from HPTN052 cohort. A5175 and A5208 did not uniformly collect hepatitis B serology. ACTG A5208 did not collect alcohol or illicit drug use.

Of the 253 pregnancies, there were 160 (63%) live births (Table 2). Of the 160 live births, 84 (53%) occurred prior to 37 weeks completed gestation and 76 (47%) were term. The overall median gestational age (GA) of live births was 35 weeks and 5 days (IQR: 35.1, 38.1). The median GA at birth among those women on a PI-based ART regimen was 36.5 weeks (IQR: 35.1, 38.1); among women on an NNRTI-based regimen was 37 weeks (IQR: 35.3, 38.1); and among women on NRTIs was 36.4 weeks (IQR: 34.4, 37.4). The median birth weight among the 68 live-born infants with a recorded birth weight was 2900 g (IQR: 2500–3400 g). The proportion of births after 20 weeks that resulted in a stillbirth was 11 (4%). Fifty-one (20%) pregnancies were recorded as spontaneous abortions and 28 (11%) were elective terminations.

**Table 2:** Pregnancy outcomes among HIV-infected women on ART prior to conception.

	Total	PI-based ART	NNRTI-based ART	NRTI-only ART
Outcome	(N = 253)	(N = 118)	(N = 127)	(N = 8)
Live births	160 (63%)	76 (64%)	79 (62%)	5 (63%)
Full term births (≥37 wks)	76 (30%)	31 (26%)	43 (34%)	2 (25%)
Preterm births (<37 wks)	84 (33%)	45 (38%)	36 (28%)	3 (38%)
Stillbirth (≥20 wks)	11 (4%)	4 (3%)	5 (4%)	2 (25%)
Spontaneous abortion	51 (20%)	27 (23%)	23 (18%)	1 (13%)
Ectopic pregnancy	3 (1%)	1 (1%)	2 (2%)	0 (0%)
Elective termination	28 (11%)	10 (8%)	18 (14%)	0 (0%)

### Associations with non-live birth

**Fig 1:**
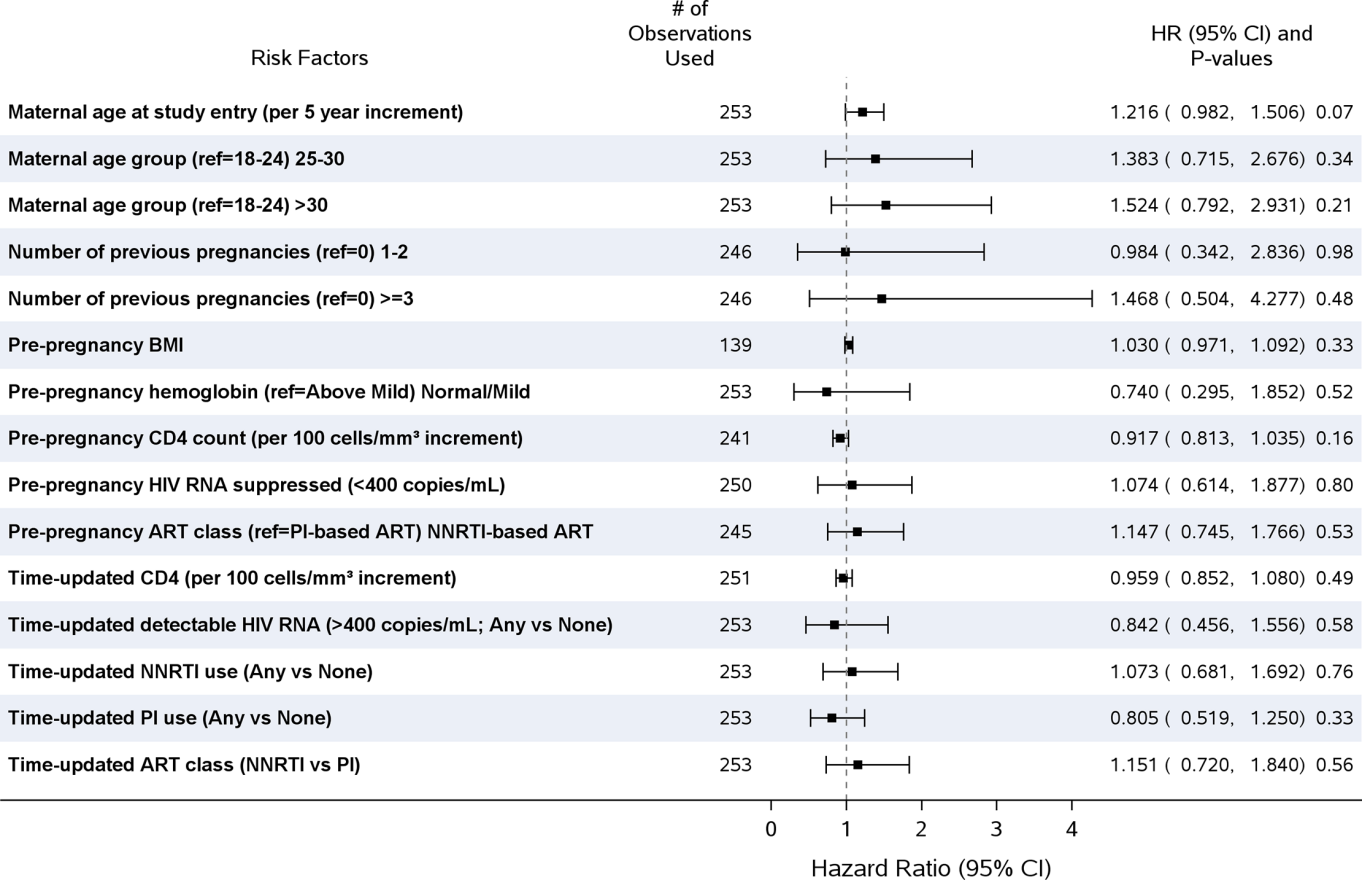
Cox proportional hazard models of time to non-live birth pregnancy outcomes (adjusted for region and parent study).

Baseline characteristics by live birth versus other birth outcome are summarized in Table 3. The median pre-pregnancy CD4+ cell count was significantly higher among women with live births: median 451 cells/mm^3^ [IQR: 321–615 cells/mm^3^] versus 399 [276–559] in non-live births; p = 0.045. Maternal age at study entry, number of prior pregnancies, pre-pregnancy BMI, pre-pregnancy hemoglobin grade, pre-pregnancy CD4+ cell count, pre-pregnancy HIV RNA suppression (< = 400 copies/mL), pre-pregnancy ART class, any detectable HIV RNA (>400 copies/mL), and any use of either an NNRTI or PI were not significantly associated with non-live birth in Cox proportional hazards regression models adjusted for region and parent study (Fig 1); there were no significant multivariable models. In a post hoc analysis where the multivariable Cox model was adjusted for region, parent study, and pre-pregnancy ART class, older age was not significantly associated with non-live birth [HR 1.25 per 5 years older; 95% CI: 0.997–1.57; p = 0.07 (Table 4)]. “A sensitivity analysis that removed 28 elective terminations was performed. Older age became significantly associated with non-live birth [HR 1.42 per 5 years older; 95% CI: 1.08–1.87; p = 0.012]”.

**Table 3:** Participant characteristics by pregnancy outcome.

			Pregnancy outcome			
		Total	Live birth	Other		
Characteristic		(N = 253)	(N = 160)	(N = 93)	p value	
Parent study	HPTN052	114	80 (70.2%)	34 (29.8%)	0.046*	
	A5208	89	55 (61.8%)	34 (38.2%)		
	A5175	50	25 (50.0%)	25 (50.0%)		
Region	Other Africa	85	58 (68.2%)	27 (31.8%)	0.09*	
	Malawi	84	59 (70.2%)	25 (29.8%)		
	South Africa	48	24 (50.0%)	24 (50.0%)		
	Asia	19	10 (52.6%)	9 (47.4%)		
	Central/South America	17	9 (52.9%)	8 (47.1%)		
Maternal age at study entry (years)	Median (IQR)	29 (26, 33)	28 (25, 32)	30 (27, 33)	0.05**	
	>30	105	61 (58.1%)	44 (41.9%)	0.20*	
	25–30	103	66 (64.1%)	37 (35.9%)		
	18–24	45	33 (73.3%)	12 (26.7%)		
Pre-pregnancy ART class	NNRTI-based ART	127	79 (62.2%)	48 (37.8%)	0.94*	
	PI-based ART	118	76 (64.4%)	42 (35.6%)		
	NRTI-only ART	8	5 (62.5%)	3 (37.5%)		
Pre-pregnancy CD4 count (cells/mm^3^)	N	241	152	89	0.045**	
	Median (IQR)	431 (304, 608)	451 (320, 615)	399 (276, 559)		
Pre-pregnancy CD8 count (cells/mm^3^)	N	123	72	51	0.92**	
	Median (IQR)	748 (550, 980)	736 (553, 958)	755 (550, 1,000)		
Pre-pregnancy HIV RNA suppressed to below 400 copies/mL	N	250	159	91	0.73*	
	Yes	205	129 (62.9%)	76 (37.1%)		
	No	45	30 (66.7%)	15 (33.3%)		
Pre-pregnancy HIV RNA among those not suppressed (log_10_copies/mL)	N	45	30	15	0.59**	
	Median (IQR)	3.8 (3.0, 4.6)	3.6 (2.8, 4.6)	3.8 (3.2, 4.7)		
Pre-pregnancy hemoglobin grade	Normal/Mild	243	155 (63.8%)	88 (36.2%)	0.59*	
	Moderate	5	2 (40.0%)	3 (60.0%)		
	Severe	5	3 (60.0%)	2 (40.0%)		
Pre-pregnancy creatinine grade	N	139	80	59	0.42*	
	Normal/Mild	138	80 (58.0%)	58 (42.0%)		
	Severe	1	0 (0.0%)	1 (100.0%)		
Pre-pregnancy body mass index (BMI, kg/m^2^)	N	139	80	59	0.07**	
	Median (IQR)	23.5 (20.5, 26.8)	23.1 (20.3, 25.6)	24.4 (20.6, 28.5)		
Pre-pregnancy hepatitis B infection	N	30	17	13	>0.99*	
	No	27	15 (55.6%)	12 (44.4%)		
	Yes	3	2 (66.7%)	1 (33.3%)		
Pre-pregnancy use of alcohol or illicit drugs	N	158	103	55	>0.99*	
	No	148	96 (64.9%)	52 (35.1%)		
	Yes	10	7 (70.0%)	3 (30.0%)		
Number of previous pregnancies	N	246	155	91	0.49*	
	0	9	5 (55.6%)	4 (44.4%)		
	1–2	128	85 (66.4%)	43 (33.6%)		
	≥3	109	65 (59.6%)	44 (40.4%)		
Number of pregnancies per woman during the study period	1	224	146 (65.2%)	78 (34.8%)	0.17*	
	2	27	13 (48.1%)	14 (51.9%)		
	3	2	1 (50.0%)	1 (50.0%)		

IQR = interquartile range

*Fisher's Exact Test; See Table 1 caption for information on missing data.

**Wilcoxon Test

**Table 4:** Cox proportional hazards models (adjusted for region, parent study, and pre-pregnancy ART class).

	Number of Observations	HR (95% CI)	p-value
Time to Non-Live Birth:	245		
Maternal age (per 5 year increment)		1.23 (0.98, 1.55)	0.07
Time to Preterm Birth:	155		
Maternal age group 25–30		2.49 (1.18, 5.56)	0.017
Maternal age group >30		1.65 (0.80, 3.40)	0.18

Analyses restricted to pre-pregnancy ART class of PI or NNRTI since few were on 3-NRTIs.

### Associations with preterm live birth

**Fig 2:**
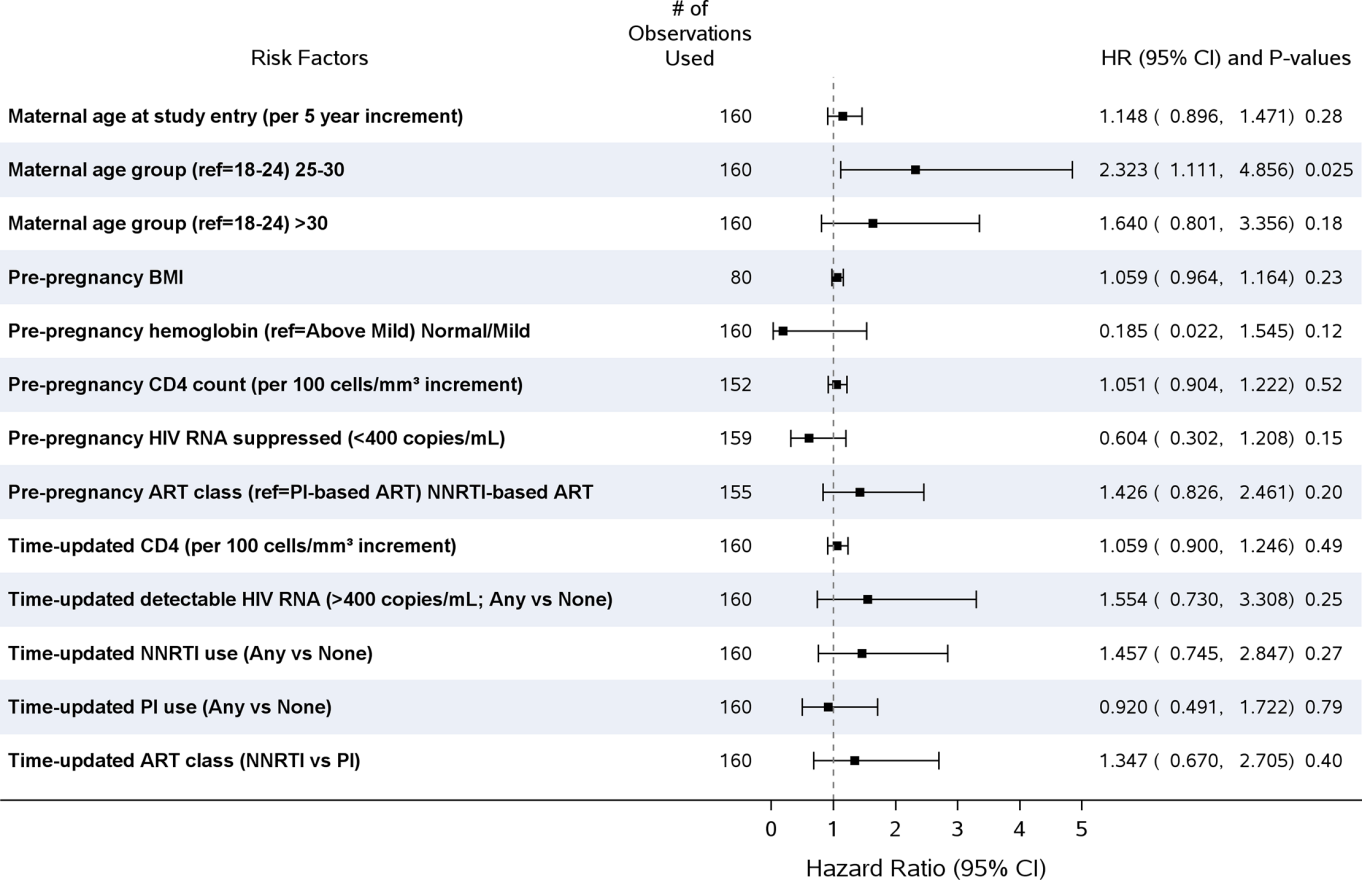
Cox proportional hazard models of time to preterm live birth pregnancy outcomes (adjusted for region and parent study).

In Cox proportional hazards regression analysis of time to preterm live birth adjusted for region and parent study, we found no factors to be significantly associated with preterm birth among women with a live birth (Fig 2); there were no significant multivariable models. In a post hoc analysis where the multivariable Cox model was adjusted for region, parent study, and pre-pregnancy ART class, maternal age 25–30 years compared to age 18–24 was significantly associated with increased hazard of preterm birth [HR 2.49; 95% CI: 1.18–5.26; p = 0.017] (Table 4).

## Discussion

In this analysis of women in resource-limited settings who conceived while taking ART in a clinical trial, adverse pregnancy outcomes were common. Only 30% of women who became pregnant delivered a live-born infant at term. Approximately half of the live-born infants were preterm. The proportion of pregnancies that ended in a stillbirth was 4%, which is considerably higher than reported stillbirth rates in other African countries,[18] and 20% of pregnancies ended in a spontaneous abortion. In understanding the results of this analysis, it is important to note that women enrolled in these clinical trials had mandatory and frequent testing for pregnancy. It is likely that the frequent pregnancy testing detected spontaneous abortions that might have otherwise been missed if women had not been followed as closely. No specific antiretroviral regimen was associated with more adverse birth outcomes.

We were surprised at the high proportion of adverse pregnancy outcomes. In the recent PROMISE trial (IMPAACT 1077BF/1077FF), which compared two PI-containing ART regimens to non-suppressive zidovudine prophylaxis, they also found high rates of adverse birth outcomes with 33.7% of pregnancies reaching the combined outcome of preterm birth, low birthweight, stillbirth, or abortion[19]. Since the PROMISE trial only enrolled women after 14 weeks gestation (median gestational age at enrollment: 26 weeks), it is likely that many of the spontaneous and elective terminations that we saw in the present analysis were not detected in PROMISE. Our rates of spontaneous abortion do not differ substantially from rates seen in the few studies that assess miscarriage rates in the general population[20].

The stillbirth numbers we observed were substantially higher than what has been reported in other African populations[18,21]; however, these other studies were not restricted to HIV-infected women on ART. Among studies of HIV-infected women on preconception ART, our stillbirth proportions remain consistent, but also unacceptably high. Chen and colleagues reported overall stillbirth rates of 4.6% among 9504 births to HIV-infected women in Botswana, but a 6.3% stillbirth rate among women who conceived on ART which is similar to what we found[11]. The etiologies of antepartum and intrapartum stillbirths are very different [22] and many of these risk factors were not available in our study or in Chen’s study; knowledge of these risk factors will impact interventions to mitigate risk of stillbirth. Maternal co-morbidities in pregnancy, such as preeclampsia and gestational hypertension, are also known risk factors for stillbirth [23] and may be higher in HIV-infected women on ART[24]. The proportion of women who delivered a preterm birth in our study was substantially higher than other studies and this may be, in part, due to lack of ultrasound dating and reliance on last menstrual period (LMP) for to determine an estimated gestational age.

It is possible that adverse birth outcomes among HIV-infected women may be related to chronic inflammation. Inflammation is known to be associated with stillbirth, preterm births, and intrauterine growth restriction[25]. Many patients who are stable on ART continue to have chronic residual immune activation, and this may be contributing to higher rates of adverse pregnancy outcomes among HIV-infected women in resource-limited settings who, until recently, have started ART later in their HIV infections.

Our study is unique because of the follow-up from before conception, monthly recording of LMP, and frequent urine pregnancy tests that allows for diagnosis of pregnancy in the first trimester, which many studies lack. This is also an improvement over simple reliance on LMP recollection and likely accounts for the high numbers of spontaneous abortions and elective terminations that we were able to capture. Another strength is the accurate recording of antiretroviral drug regimens and the switching of regimens, as well as discontinuation of regimens; we observed no impact of certain antiretrovirals on adverse birth outcomes.

Because the parent studies were designed to measure HIV outcomes and not obstetrical outcomes, our analysis has several important limitations. A reliable gestational age determination is critical to assessing pregnancy outcomes; dating based solely on LMP is notoriously inaccurate[26]. However, we note that all three protocols assessed LMP at each visit, a practice that we expect would make patient recollection more reliable. Additionally, although relying on dating of pregnancies by LMP alone is a weakness, there is no reason to think that the estimated gestational age at delivery would be systematically biased one way or another for the overall outcomes. An additional weakness is the small sample size and missing birthweight data that prevented us from being able to detect even moderate differences in birth outcomes or birthweights among specific antiretroviral regimens. We would have liked to have collected more detailed information on the types of preterm birth (spontaneous versus indicated) and stillbirth (pre-labor/macerated versus intrapartum/fresh) in our cohort. The underlying etiologies for spontaneous preterm birth and indicated preterm birth differ substantially and must be understood with regard to HIV-infected pregnant women who are on ART before making conclusions about causality[27]. Finally, because of the multiple inclusion and exclusion criteria for the three included studies, our results may not be generalizable to a larger population of pregnant HIV infected women.

Since the introduction of Option B+ in many resource-limited settings, the number of infants who are perinatally-infected with HIV has fallen precipitously, but the relationship between HIV infection, timing of initiation of ART, and actual ART regimens on adverse pregnancy outcomes remain elusive, for the most part, because of the lack of well-designed studies that are focused on pregnancy outcomes as a primary outcome.

## Conclusion

In our study, adverse pregnancy outcomes including stillbirth and preterm birth were high among an HIV-infected population of women who were on ART at the time of conception. Studies that more accurately capture gestational age at birth, indications for preterm births, and maternal birth history, while examining commonly used ART regimens, timing of ART administration in the pre- and post-conception period, as well as immune status at the time of initiation of ART are urgently needed to inform the management of HIV infection in pregnancy.

## Supporting information

S1 Table List of ethics boards that approved the studies. (DOC)

S2 Table Deidentified study data file. (XLSX)
