# Supplementary material for: Pregnancy outcomes among HIV-infected women who conceived on antiretroviral therapy
Source: PLoS One. 2018 Jul 18;13(7):e0199555. doi: 10.1371/journal.pone.0199555 (PMC6051581; doi:10.1371/journal.pone.0199555)
Supplement: S1 Table — (DOC) [file pone.0199555.s001.doc]

IRBs/ECs and Other Regulatory Bodies by Site_HPTN_Site	Affiliated IRBs/ECs and Regulatory Bodies	
Porto Alegre, Brazil	UCLA Office for Protection of Research Subjects: Medical Institutional Review BoardBrazil Ministério da Saúde: CONEP: Comissão Nacional de Ética em PesquisaGerencia de Ensino e Pesuisa: Comitê de Ética em Pesquisa do Grupo Hospitalar Conceição-GHC	
Rio de Janeiro, Brazil	UCLA Office for Protection of Research Subjects: Medical InstitutionalReview BoardInstituto de Pesquisa Clínica Evandro Chagas: Comitê de Ética em PesquisaBrazil Ministério da Saúde: CONEP: Comissão Nacional de Ética em PesquisaGrupo Hospitalar Conceição-GHC: Comitê de Ética em Pesquisa	
Chiang Mai, Thailand	Johns Hopkins Bloomberg School of Public Health Institutional Review BoardsResearch Ethics Committee, Faculty of Medicine, Chiang Mai UniversityEthical Review Committee for Research in Human Subjects Ministry of Public Health, Thailand	
Kericho, Kenya	Kenya Medical Research Institute: KEMRI National Ethical Review CommitteeWalter Reed Army Institute of Research Kenya National Pharmacy and Poisons Board (PPB)	
Eldoret, Kenya	AMPATH Center at Moi University Ethics CommitteeIndiana University Institutional Review BoardKenya National Pharmacy and Poisons Board (PPB)	
Kisumu, Kenya	Centers for Disease Control Institutional Review BoardKenya National Pharmacy and Poisons Board (PPB)	
Site	Affiliated IRBs/RECs and Regulatory Bodies	
Harare, Zimbabwe	University of California at San Francisco: Committee on Human Research, Office of Research AdministrationMedical Research Council of Zimbabwe: Medical Research Council   of Zimbabwe (MRCZ) Institutional Review BoardMedicines Control Authority of Zimbabwe (MCAZ)Research Council of Zimbabwe (RCZ)	
Blantyre, Malawi	University of Malawi College of Medicine: College of MedicineResearch & Ethics Committee (COMREC)Johns Hopkins University Bloomberg School of Public Health: Institutional Review Board	
Lusaka, Zambia	Zambian Research Ethics CommitteeUniversity of Alabama at Birmingham, Institutional Review Board	
Kampala, Uganda	Uganda National Council for Science and Technology Case Western Reserve University Institutional Review Board	
Lilongwe, Malawi	Malawi Ministry of Health & Population: National Health Sciences Research CommitteeUniversity of North Carolina School of Medicine Institutional Review Board	
Gaborone, Botswana	Botswana Ministry of Health: Health Research and DevelopmentCommitteeHarvard School of Public Health: Human Subjects Committee	
Johannesburg, South Africa	University of Witwatersrand: Human Research Ethics Committee: MedicalMedicines Control Council (South Africa)	
Soweto, South Africa	University of Witwatersrand: Human Research Ethics Committee: MedicalMedicines Control Council (South Africa)	
